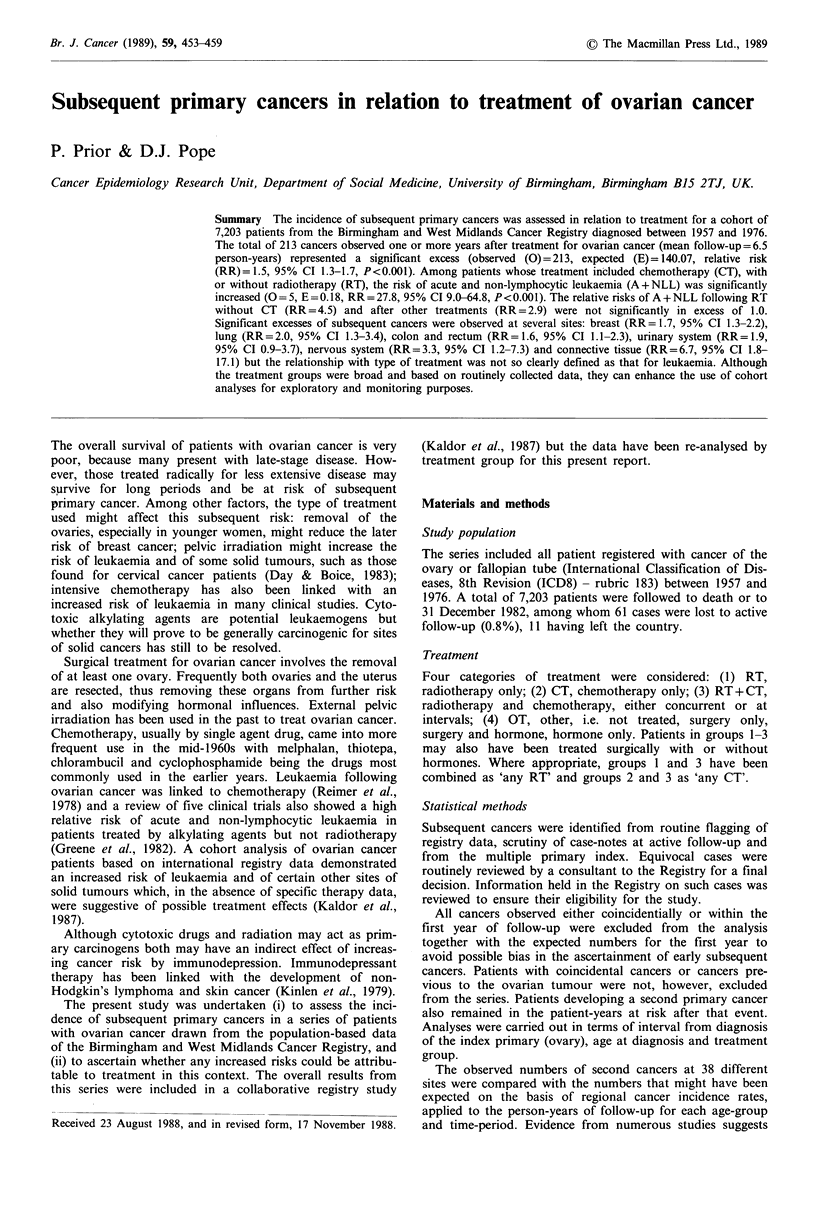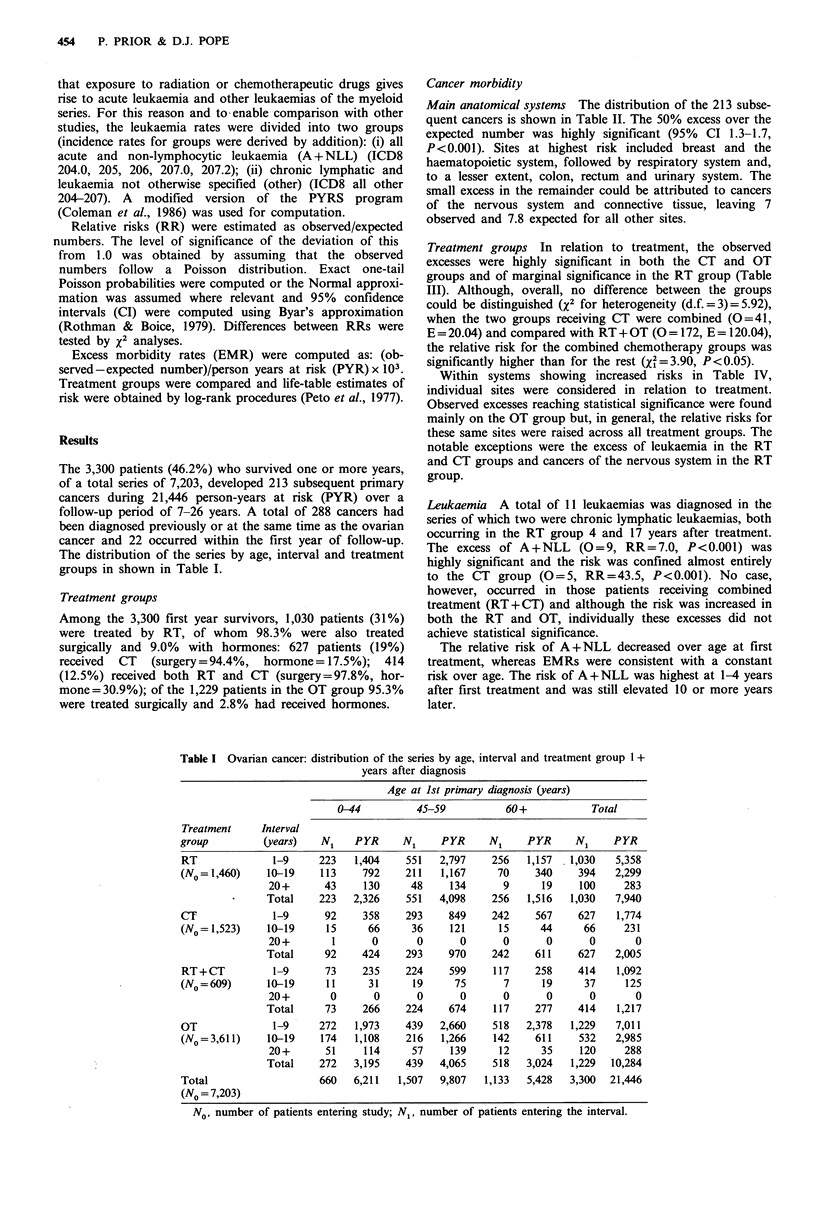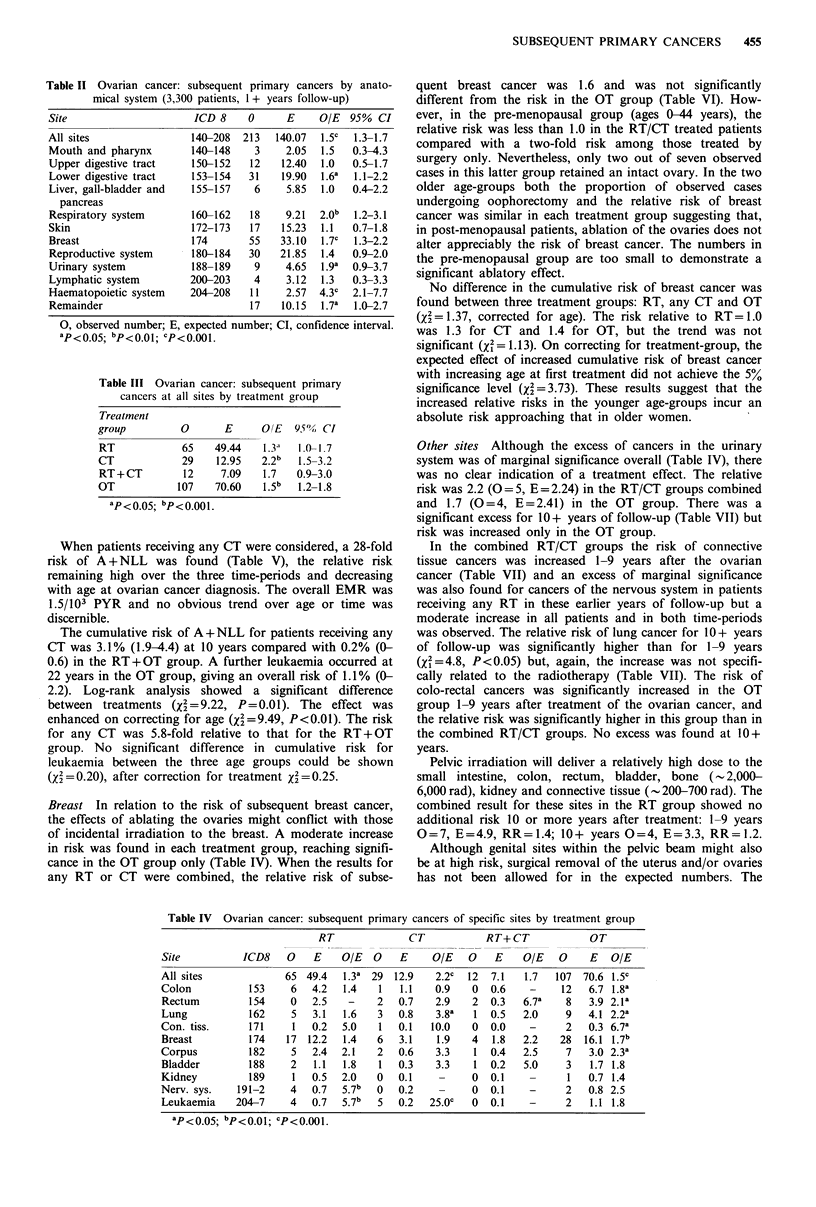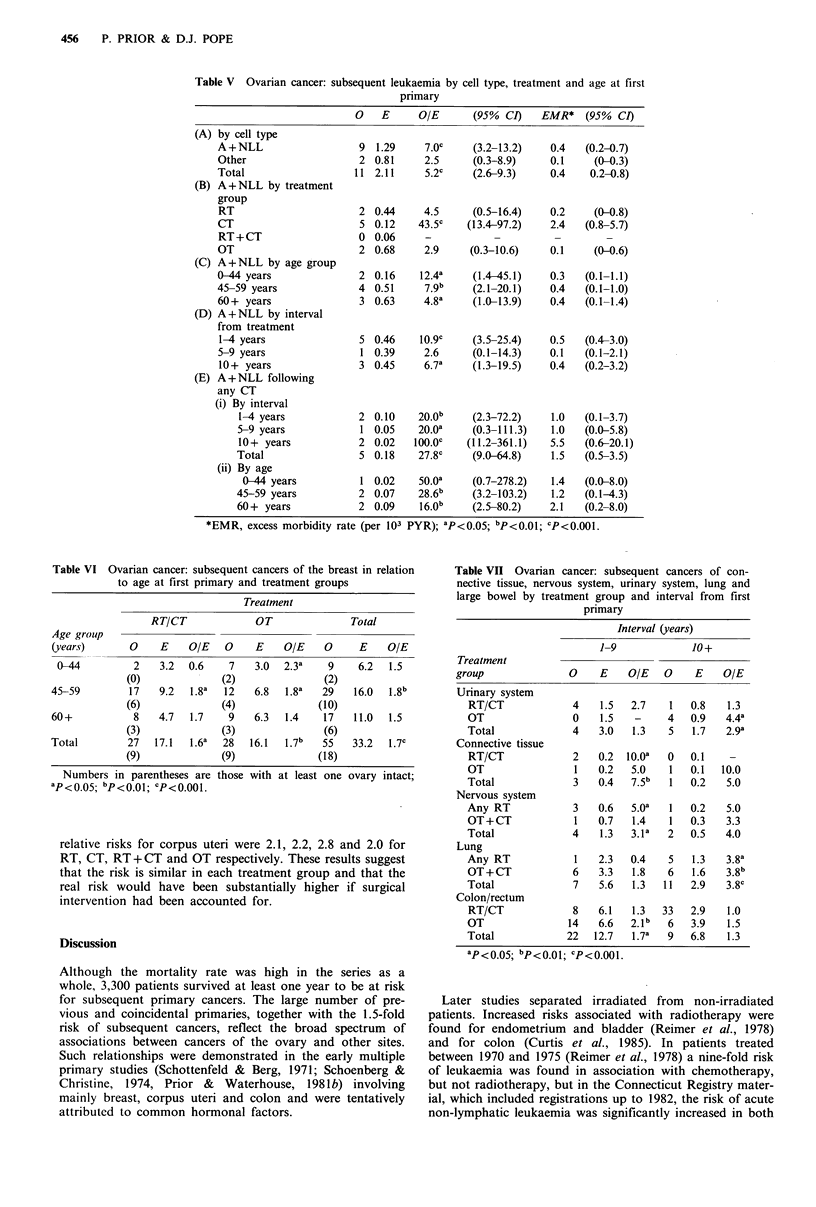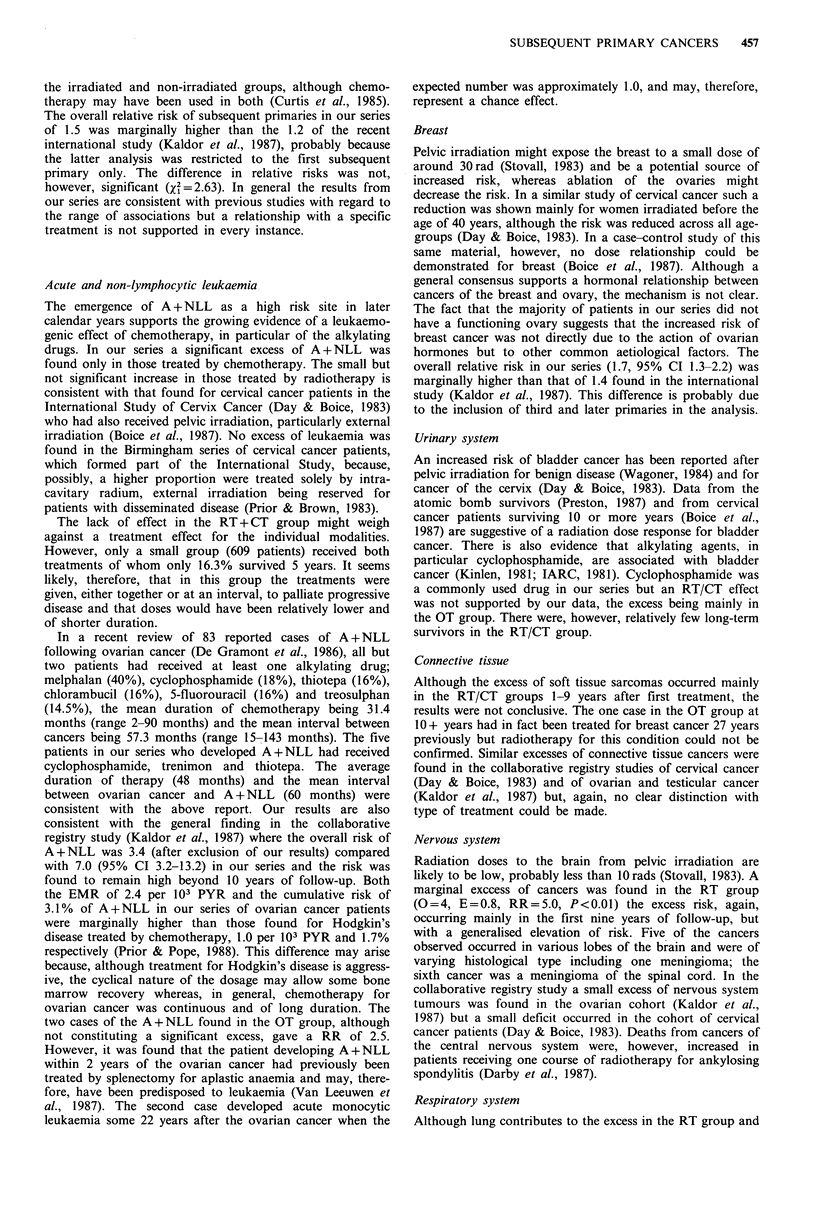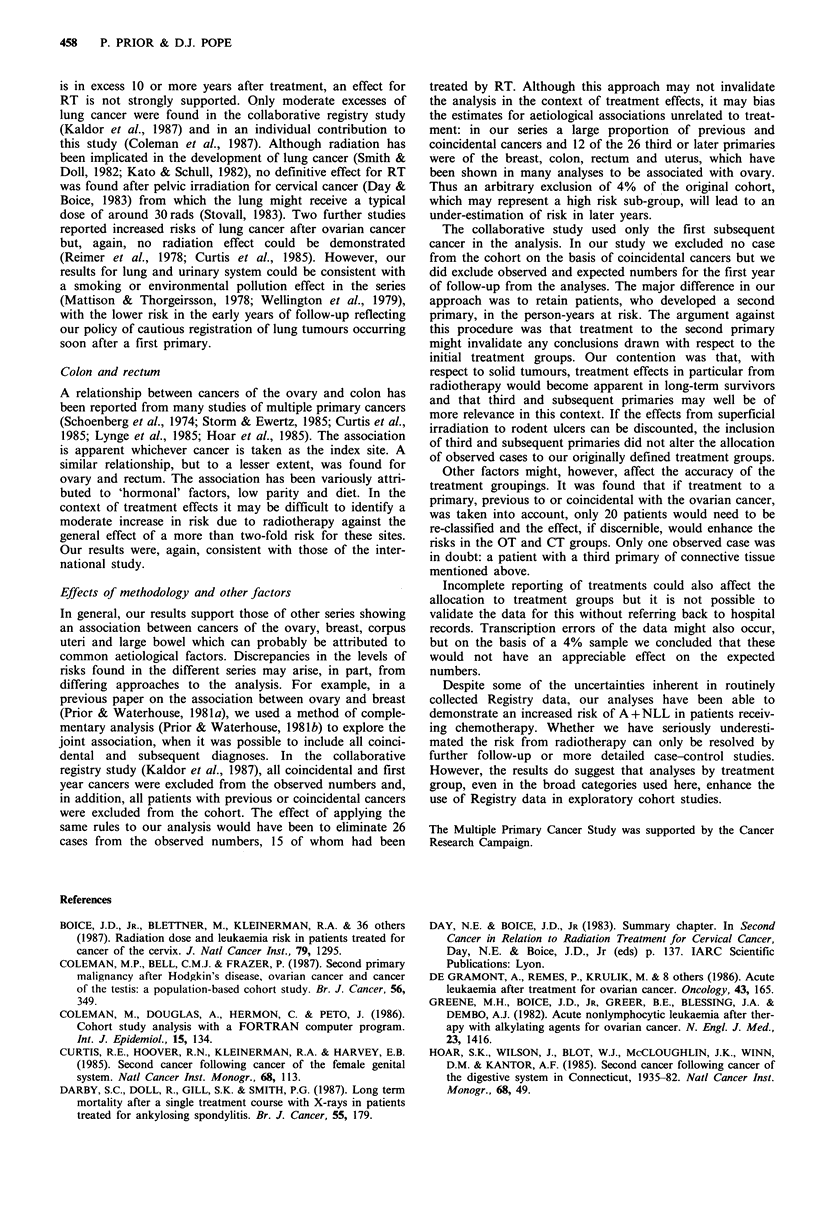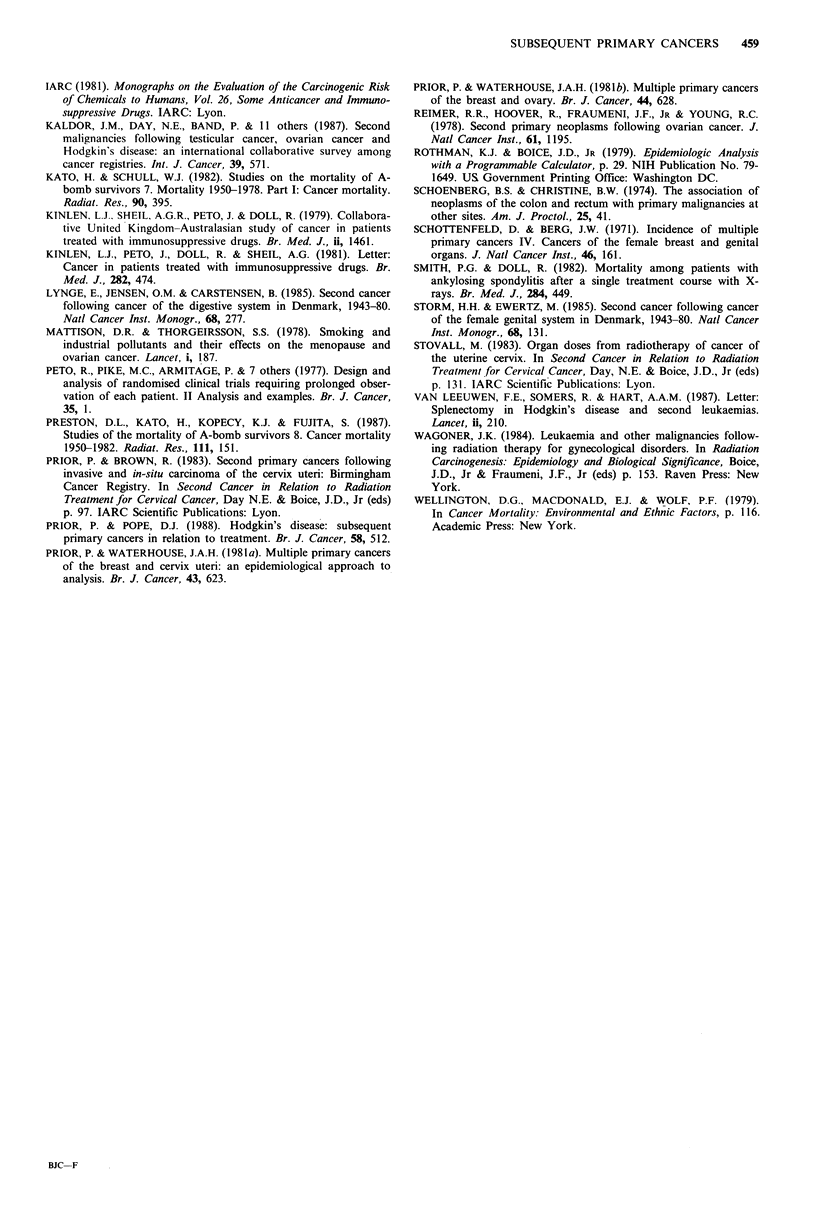# Subsequent primary cancers in relation to treatment of ovarian cancer.

**DOI:** 10.1038/bjc.1989.93

**Published:** 1989-03

**Authors:** P. Prior, D. J. Pope

**Affiliations:** Cancer Epidemiology Research Unit, Department of Social Medicine, University of Birmingham, UK.

## Abstract

The incidence of subsequent primary cancers was assessed in relation to treatment for a cohort of 7,203 patients from the Birmingham and West Midlands Cancer Registry diagnosed between 1957 and 1976. The total of 213 cancers observed one or more years after treatment for ovarian cancer (mean follow-up = 6.5 person-years) represented a significant excess (observed (O) = 213, expected (E) = 140.07, relative risk (RR) = 1.5, 95% CI 1.3-1.7, P less than 0.001). Among patients whose treatment included chemotherapy (CT), with or without radiotherapy (RT), the risk of acute and non-lymphocytic leukaemia (A + NLL) was significantly increased (O = 5, E = 0.18, RR = 27.8, 95% CI 9.0-64.8, P less than 0.001). The relative risks of A + NLL following RT without CT (RR = 4.5) and after other treatments (RR = 2.9) were not significantly in excess of 1.0. Significant excesses of subsequent cancers were observed at several sites: breast (RR = 1.7, 95% CI 1.3-2.2), lung (RR = 2.0, 95% CI 1.3-3.4), colon and rectum (RR = 1.6, 95% CI 1.1-2.3), urinary system (RR = 1.9, 95% CI 0.9-3.7), nervous system (RR = 3.3, 95% CI 1.2-7.3) and connective tissue (RR = 6.7, 95% CI 1.8-17.1) but the relationship with type of treatment was not so clearly defined as that for leukaemia. Although the treatment groups were broad and based on routinely collected data, they can enhance the use of cohort analyses for exploratory and monitoring purposes.


*Br. J. Cancer* (1989), **59**, 453–459 © The Macmillan Press Ltd., 1989

### Subsequent primary cancers in relation to treatment of ovarian cancer

P. Prior & D.J. Pope

*Cancer Epidemiology Research Unit, Department of Social Medicine, University of Birmingham, Birmingham B15 2TJ, UK.*

**Summary** The incidence of subsequent primary cancers was assessed in relation to treatment for a cohort of 7,203 patients from the Birmingham and West Midlands Cancer Registry diagnosed between 1957 and 1976. The total of 213 cancers observed one or more years after treatment for ovarian cancer (mean follow-up = 6.5 person-years) represented a significant excess (observed (O) = 213, expected (E) = 140.07, relative risk (RR) = 1.5, 95% CI 1.3–1.7, $P<0.001$). Among patients whose treatment included chemotherapy (CT), with or without radiotherapy (RT), the risk of acute and non-lymphocytic leukaemia (A + NLL) was significantly increased (O = 5, E = 0.18, RR = 27.8, 95% CI 9.0–64.8, $P<0.001$). The relative risks of A + NLL following RT without CT (RR = 4.5) and after other treatments (RR = 2.9) were not significantly in excess of 1.0. Significant excesses of subsequent cancers were observed at several sites: breast (RR = 1.7, 95% CI 1.3–2.2), lung (RR = 2.0, 95% CI 1.3–3.4), colon and rectum (RR = 1.6, 95% CI 1.1–2.3), urinary system (RR = 1.9, 95% CI 0.9–3.7), nervous system (RR = 3.3, 95% CI 1.2–7.3) and connective tissue (RR = 6.7, 95% CI 1.8–17.1) but the relationship with type of treatment was not so clearly defined as that for leukaemia. Although the treatment groups were broad and based on routinely collected data, they can enhance the use of cohort analyses for exploratory and monitoring purposes.

The overall survival of patients with ovarian cancer is very poor, because many present with late-stage disease. However, those treated radically for less extensive disease may survive for long periods and be at risk of subsequent primary cancer. Among other factors, the type of treatment used might affect this subsequent risk: removal of the ovaries, especially in younger women, might reduce the later risk of breast cancer; pelvic irradiation might increase the risk of leukaemia and of some solid tumours, such as those found for cervical cancer patients (Day & Boice, 1983); intensive chemotherapy has also been linked with an increased risk of leukaemia in many clinical studies. Cytotoxic alkylating agents are potential leukaemogens but whether they will prove to be generally carcinogenic for sites of solid cancers has still to be resolved.

Surgical treatment for ovarian cancer involves the removal of at least one ovary. Frequently both ovaries and the uterus are resected, thus removing these organs from further risk and also modifying hormonal influences. External pelvic irradiation has been used in the past to treat ovarian cancer. Chemotherapy, usually by single agent drug, came into more frequent use in the mid-1960s with melphalan, thiotepa, chlorambucil and cyclophosphamide being the drugs most commonly used in the earlier years. Leukaemia following ovarian cancer was linked to chemotherapy (Reimer *et al.*, 1978) and a review of five clinical trials also showed a high relative risk of acute and non-lymphocytic leukaemia in patients treated by alkylating agents but not radiotherapy (Greene *et al.*, 1982). A cohort analysis of ovarian cancer patients based on international registry data demonstrated an increased risk of leukaemia and of certain other sites of solid tumours which, in the absence of specific therapy data, were suggestive of possible treatment effects (Kaldor *et al.*, 1987).

Although cytotoxic drugs and radiation may act as primary carcinogens both may have an indirect effect of increasing cancer risk by immunodepression. Immunodepressant therapy has been linked with the development of non-Hodgkin's lymphoma and skin cancer (Kinlen *et al.*, 1979).

The present study was undertaken (i) to assess the incidence of subsequent primary cancers in a series of patients with ovarian cancer drawn from the population-based data of the Birmingham and West Midlands Cancer Registry, and (ii) to ascertain whether any increased risks could be attributable to treatment in this context. The overall results from this series were included in a collaborative registry study (Kaldor *et al.*, 1987) but the data have been re-analysed by treatment group for this present report.

Received 23 August 1988, and in revised form, 17 November 1988.

### Materials and methods

### *Study population*

The series included all patient registered with cancer of the ovary or fallopian tube (International Classification of Diseases, 8th Revision (ICD8) – rubric 183) between 1957 and 1976. A total of 7,203 patients were followed to death or to 31 December 1982, among whom 61 cases were lost to active follow-up (0.8%), 11 having left the country.

### *Treatment*

Four categories of treatment were considered: (1) RT, radiotherapy only; (2) CT, chemotherapy only; (3) RT + CT, radiotherapy and chemotherapy, either concurrent or at intervals; (4) OT, other, i.e. not treated, surgery only, surgery and hormone, hormone only. Patients in groups 1–3 may also have been treated surgically with or without hormones. Where appropriate, groups 1 and 3 have been combined as 'any RT' and groups 2 and 3 as 'any CT'.

### *Statistical methods*

Subsequent cancers were identified from routine flagging of registry data, scrutiny of case-notes at active follow-up and from the multiple primary index. Equivocal cases were routinely reviewed by a consultant to the Registry for a final decision. Information held in the Registry on such cases was reviewed to ensure their eligibility for the study.

All cancers observed either coincidentially or within the first year of follow-up were excluded from the analysis together with the expected numbers for the first year to avoid possible bias in the ascertainment of early subsequent cancers. Patients with coincidental cancers or cancers previous to the ovarian tumour were not, however, excluded from the series. Patients developing a second primary cancer also remained in the patient-years at risk after that event. Analyses were carried out in terms of interval from diagnosis of the index primary (ovary), age at diagnosis and treatment group.

The observed numbers of second cancers at 38 different sites were compared with the numbers that might have been expected on the basis of regional cancer incidence rates, applied to the person-years of follow-up for each age-group and time-period. Evidence from numerous studies suggests that exposure to radiation or chemotherapeutic drugs gives rise to acute leukaemia and other leukaemias of the myeloid series. For this reason and to enable comparison with other studies, the leukaemia rates were divided into two groups (incidence rates for groups were derived by addition): (i) all acute and non-lymphocytic leukaemia (A+NLL) (ICD8 204.0, 205, 206, 207.0, 207.2); (ii) chronic lymphatic and leukaemia not otherwise specified (other) (ICD8 all other 204–207). A modified version of the PYRS program (Coleman *et al.*, 1986) was used for computation.

Relative risks (RR) were estimated as observed/expected numbers. The level of significance of the deviation of this from 1.0 was obtained by assuming that the observed numbers follow a Poisson distribution. Exact one-tail Poisson probabilities were computed or the Normal approximation was assumed where relevant and 95% confidence intervals (CI) were computed using Byar's approximation (Rothman & Boice, 1979). Differences between RRs were tested by $\chi^2$ analyses.

Excess morbidity rates (EMR) were computed as: (observed − expected number)/person years at risk (PYR) $\times 10^3$. Treatment groups were compared and life-table estimates of risk were obtained by log-rank procedures (Peto *et al.*, 1977).

### Results

The 3,300 patients (46.2%) who survived one or more years, of a total series of 7,203, developed 213 subsequent primary cancers during 21,446 person-years at risk (PYR) over a follow-up period of 7–26 years. A total of 288 cancers had been diagnosed previously or at the same time as the ovarian cancer and 22 occurred within the first year of follow-up. The distribution of the series by age, interval and treatment groups in shown in Table I.

### *Treatment groups*

Among the 3,300 first year survivors, 1,030 patients (31%) were treated by RT, of whom 98.3% were also treated surgically and 9.0% with hormones: 627 patients (19%) received CT (surgery = 94.4%, hormone = 17.5%); 414 (12.5%) received both RT and CT (surgery = 97.8%, hormone = 30.9%); of the 1,229 patients in the OT group 95.3% were treated surgically and 2.8% had received hormones.

### *Cancer morbidity*

*Main anatomical systems* The distribution of the 213 subsequent cancers is shown in Table II. The 50% excess over the expected number was highly significant (95% CI 1.3–1.7, $P<0.001$). Sites at highest risk included breast and the haematopoietic system, followed by respiratory system and, to a lesser extent, colon, rectum and urinary system. The small excess in the remainder could be attributed to cancers of the nervous system and connective tissue, leaving 7 observed and 7.8 expected for all other sites.

*Treatment groups* In relation to treatment, the observed excesses were highly significant in both the CT and OT groups and of marginal significance in the RT group (Table III). Although, overall, no difference between the groups could be distinguished ($\chi^2$ for heterogeneity (d.f. = 3) = 5.92), when the two groups receiving CT were combined (O = 41, E = 20.04) and compared with RT+OT (O = 172, E = 120.04), the relative risk for the combined chemotherapy groups was significantly higher than for the rest ($\chi^2_1 = 3.90$, $P<0.05$).

Within systems showing increased risks in Table IV, individual sites were considered in relation to treatment. Observed excesses reaching statistical significance were found mainly on the OT group but, in general, the relative risks for these same sites were raised across all treatment groups. The notable exceptions were the excess of leukaemia in the RT and CT groups and cancers of the nervous system in the RT group.

*Leukaemia* A total of 11 leukaemias was diagnosed in the series of which two were chronic lymphatic leukaemias, both occurring in the RT group 4 and 17 years after treatment. The excess of A+NLL (O = 9, RR = 7.0, $P<0.001$) was highly significant and the risk was confined almost entirely to the CT group (O = 5, RR = 43.5, $P<0.001$). No case, however, occurred in those patients receiving combined treatment (RT+CT) and although the risk was increased in both the RT and OT, individually these excesses did not achieve statistical significance.

The relative risk of A+NLL decreased over age at first treatment, whereas EMRs were consistent with a constant risk over age. The risk of A+NLL was highest at 1–4 years after first treatment and was still elevated 10 or more years later.

**Table I** Ovarian cancer: distribution of the series by age, interval and treatment group 1+ years after diagnosis

| | | *Age at 1st primary diagnosis (years)* | | | | | | | |
|---|---|---|---|---|---|---|---|---|---|
| | | *0–44* | | *45–59* | | *60+* | | *Total* | |
| *Treatment group* | *Interval (years)* | $N_1$ | *PYR* | $N_1$ | *PYR* | $N_1$ | *PYR* | $N_1$ | *PYR* |
| RT | 1–9 | 223 | 1,404 | 551 | 2,797 | 256 | 1,157 | 1,030 | 5,358 |
| ($N_0$ = 1,460) | 10–19 | 113 | 792 | 211 | 1,167 | 70 | 340 | 394 | 2,299 |
| | 20+ | 43 | 130 | 48 | 134 | 9 | 19 | 100 | 283 |
| | Total | 223 | 2,326 | 551 | 4,098 | 256 | 1,516 | 1,030 | 7,940 |
| CT | 1–9 | 92 | 358 | 293 | 849 | 242 | 567 | 627 | 1,774 |
| ($N_0$ = 1,523) | 10–19 | 15 | 66 | 36 | 121 | 15 | 44 | 66 | 231 |
| | 20+ | 1 | 0 | 0 | 0 | 0 | 0 | 0 | 0 |
| | Total | 92 | 424 | 293 | 970 | 242 | 611 | 627 | 2,005 |
| RT+CT | 1–9 | 73 | 235 | 224 | 599 | 117 | 258 | 414 | 1,092 |
| ($N_0$ = 609) | 10–19 | 11 | 31 | 19 | 75 | 7 | 19 | 37 | 125 |
| | 20+ | 0 | 0 | 0 | 0 | 0 | 0 | 0 | 0 |
| | Total | 73 | 266 | 224 | 674 | 117 | 277 | 414 | 1,217 |
| OT | 1–9 | 272 | 1,973 | 439 | 2,660 | 518 | 2,378 | 1,229 | 7,011 |
| ($N_0$ = 3,611) | 10–19 | 174 | 1,108 | 216 | 1,266 | 142 | 611 | 532 | 2,985 |
| | 20+ | 51 | 114 | 57 | 139 | 12 | 35 | 120 | 288 |
| | Total | 272 | 3,195 | 439 | 4,065 | 518 | 3,024 | 1,229 | 10,284 |
| Total ($N_0$ = 7,203) | | 660 | 6,211 | 1,507 | 9,807 | 1,133 | 5,428 | 3,300 | 21,446 |

$N_0$, number of patients entering study; $N_1$, number of patients entering the interval.

**Table II** Ovarian cancer: subsequent primary cancers by anatomical system (3,300 patients, 1+ years follow-up)

| *Site* | *ICD 8* | *O* | *E* | *O/E* | *95% CI* |
|---|---|---|---|---|---|
| All sites | 140–208 | 213 | 140.07 | $1.5^c$ | 1.3–1.7 |
| Mouth and pharynx | 140–148 | 3 | 2.05 | 1.5 | 0.3–4.3 |
| Upper digestive tract | 150–152 | 12 | 12.40 | 1.0 | 0.5–1.7 |
| Lower digestive tract | 153–154 | 31 | 19.90 | $1.6^a$ | 1.1–2.2 |
| Liver, gall-bladder and pancreas | 155–157 | 6 | 5.85 | 1.0 | 0.4–2.2 |
| Respiratory system | 160–162 | 18 | 9.21 | $2.0^b$ | 1.2–3.1 |
| Skin | 172–173 | 17 | 15.23 | 1.1 | 0.7–1.8 |
| Breast | 174 | 55 | 33.10 | $1.7^c$ | 1.3–2.2 |
| Reproductive system | 180–184 | 30 | 21.85 | 1.4 | 0.9–2.0 |
| Urinary system | 188–189 | 9 | 4.65 | $1.9^a$ | 0.9–3.7 |
| Lymphatic system | 200–203 | 4 | 3.12 | 1.3 | 0.3–3.3 |
| Haematopoietic system | 204–208 | 11 | 2.57 | $4.3^c$ | 2.1–7.7 |
| Remainder | | 17 | 10.15 | $1.7^a$ | 1.0–2.7 |

O, observed number; E, expected number; CI, confidence interval. $^aP<0.05$; $^bP<0.01$; $^cP<0.001$.

**Table III** Ovarian cancer: subsequent primary cancers at all sites by treatment group

| *Treatment group* | *O* | *E* | *O/E* | *95% CI* |
|---|---|---|---|---|
| RT | 65 | 49.44 | $1.3^a$ | 1.0–1.7 |
| CT | 29 | 12.95 | $2.2^b$ | 1.5–3.2 |
| RT+CT | 12 | 7.09 | 1.7 | 0.9–3.0 |
| OT | 107 | 70.60 | $1.5^b$ | 1.2–1.8 |

$^aP<0.05$; $^bP<0.001$.

When patients receiving any CT were considered, a 28-fold risk of A+NLL was found (Table V), the relative risk remaining high over the three time-periods and decreasing with age at ovarian cancer diagnosis. The overall EMR was $1.5/10^3$ PYR and no obvious trend over age or time was discernible.

The cumulative risk of A+NLL for patients receiving any CT was 3.1% (1.9–4.4) at 10 years compared with 0.2% (0–0.6) in the RT+OT group. A further leukaemia occurred at 22 years in the OT group, giving an overall risk of 1.1% (0–2.2). Log-rank analysis showed a significant difference between treatments ($\chi^2_2=9.22$, $P=0.01$). The effect was enhanced on correcting for age ($\chi^2_2=9.49$, $P<0.01$). The risk for any CT was 5.8-fold relative to that for the RT+OT group. No significant difference in cumulative risk for leukaemia between the three age groups could be shown ($\chi^2_2=0.20$), after correction for treatment $\chi^2_2=0.25$.

*Breast* In relation to the risk of subsequent breast cancer, the effects of ablating the ovaries might conflict with those of incidental irradiation to the breast. A moderate increase in risk was found in each treatment group, reaching significance in the OT group only (Table IV). When the results for any RT or CT were combined, the relative risk of subsequent breast cancer was 1.6 and was not significantly different from the risk in the OT group (Table VI). However, in the pre-menopausal group (ages 0–44 years), the relative risk was less than 1.0 in the RT/CT treated patients compared with a two-fold risk among those treated by surgery only. Nevertheless, only two out of seven observed cases in this latter group retained an intact ovary. In the two older age-groups both the proportion of observed cases undergoing oophorectomy and the relative risk of breast cancer was similar in each treatment group suggesting that, in post-menopausal patients, ablation of the ovaries does not alter appreciably the risk of breast cancer. The numbers in the pre-menopausal group are too small to demonstrate a significant ablatory effect.

No difference in the cumulative risk of breast cancer was found between three treatment groups: RT, any CT and OT ($\chi^2_2=1.37$, corrected for age). The risk relative to RT=1.0 was 1.3 for CT and 1.4 for OT, but the trend was not significant ($\chi^2_1=1.13$). On correcting for treatment-group, the expected effect of increased cumulative risk of breast cancer with increasing age at first treatment did not achieve the 5% significance level ($\chi^2_2=3.73$). These results suggest that the increased relative risks in the younger age-groups incur an absolute risk approaching that in older women.

*Other sites* Although the excess of cancers in the urinary system was of marginal significance overall (Table IV), there was no clear indication of a treatment effect. The relative risk was 2.2 (O=5, E=2.24) in the RT/CT groups combined and 1.7 (O=4, E=2.41) in the OT group. There was a significant excess for 10+ years of follow-up (Table VII) but risk was increased only in the OT group.

In the combined RT/CT groups the risk of connective tissue cancers was increased 1–9 years after the ovarian cancer (Table VII) and an excess of marginal significance was also found for cancers of the nervous system in patients receiving any RT in these earlier years of follow-up but a moderate increase in all patients and in both time-periods was observed. The relative risk of lung cancer for 10+ years of follow-up was significantly higher than for 1–9 years ($\chi^2_1=4.8$, $P<0.05$) but, again, the increase was not specifically related to the radiotherapy (Table VII). The risk of colo-rectal cancers was significantly increased in the OT group 1–9 years after treatment of the ovarian cancer, and the relative risk was significantly higher in this group than in the combined RT/CT groups. No excess was found at 10+ years.

Pelvic irradiation will deliver a relatively high dose to the small intestine, colon, rectum, bladder, bone (~2,000–6,000 rad), kidney and connective tissue (~200–700 rad). The combined result for these sites in the RT group showed no additional risk 10 or more years after treatment: 1–9 years O=7, E=4.9, RR=1.4; 10+ years O=4, E=3.3, RR=1.2.

Although genital sites within the pelvic beam might also be at high risk, surgical removal of the uterus and/or ovaries has not been allowed for in the expected numbers. The

**Table IV** Ovarian cancer: subsequent primary cancers of specific sites by treatment group

| | | *RT* | | | *CT* | | | *RT+CT* | | | *OT* | | |
|---|---|---|---|---|---|---|---|---|---|---|---|---|---|
| *Site* | *ICD8* | *O* | *E* | *O/E* | *O* | *E* | *O/E* | *O* | *E* | *O/E* | *O* | *E* | *O/E* |
| All sites | | 65 | 49.4 | $1.3^a$ | 29 | 12.9 | $2.2^c$ | 12 | 7.1 | 1.7 | 107 | 70.6 | $1.5^c$ |
| Colon | 153 | 6 | 4.2 | 1.4 | 1 | 1.1 | 0.9 | 0 | 0.6 | – | 12 | 6.7 | $1.8^a$ |
| Rectum | 154 | 0 | 2.5 | – | 2 | 0.7 | 2.9 | 2 | 0.3 | $6.7^a$ | 8 | 3.9 | $2.1^a$ |
| Lung | 162 | 5 | 3.1 | 1.6 | 3 | 0.8 | $3.8^a$ | 1 | 0.5 | 2.0 | 9 | 4.1 | $2.2^a$ |
| Con. tiss. | 171 | 1 | 0.2 | 5.0 | 1 | 0.1 | 10.0 | 0 | 0.0 | – | 2 | 0.3 | $6.7^a$ |
| Breast | 174 | 17 | 12.2 | 1.4 | 6 | 3.1 | 1.9 | 4 | 1.8 | 2.2 | 28 | 16.1 | $1.7^b$ |
| Corpus | 182 | 5 | 2.4 | 2.1 | 2 | 0.6 | 3.3 | 1 | 0.4 | 2.5 | 7 | 3.0 | $2.3^a$ |
| Bladder | 188 | 2 | 1.1 | 1.8 | 1 | 0.3 | 3.3 | 1 | 0.2 | 5.0 | 3 | 1.7 | 1.8 |
| Kidney | 189 | 1 | 0.5 | 2.0 | 0 | 0.1 | – | 0 | 0.1 | – | 1 | 0.7 | 1.4 |
| Nerv. sys. | 191–2 | 4 | 0.7 | $5.7^b$ | 0 | 0.2 | – | 0 | 0.1 | – | 2 | 0.8 | 2.5 |
| Leukaemia | 204–7 | 4 | 0.7 | $5.7^b$ | 5 | 0.2 | $25.0^c$ | 0 | 0.1 | – | 2 | 1.1 | 1.8 |

$^aP<0.05$; $^bP<0.01$; $^cP<0.001$.

**Table V** Ovarian cancer: subsequent leukaemia by cell type, treatment and age at first primary

| | *O* | *E* | *O/E* | *(95% CI)* | *EMR** | *(95% CI)* |
|---|---|---|---|---|---|---|
| (A) by cell type | | | | | | |
| A+NLL | 9 | 1.29 | 7.0[c] | (3.2–13.2) | 0.4 | (0.2–0.7) |
| Other | 2 | 0.81 | 2.5 | (0.3–8.9) | 0.1 | (0–0.3) |
| Total | 11 | 2.11 | 5.2[c] | (2.6–9.3) | 0.4 | 0.2–0.8) |
| (B) A+NLL by treatment group | | | | | | |
| RT | 2 | 0.44 | 4.5 | (0.5–16.4) | 0.2 | (0–0.8) |
| CT | 5 | 0.12 | 43.5[c] | (13.4–97.2) | 2.4 | (0.8–5.7) |
| RT+CT | 0 | 0.06 | – | – | – | – |
| OT | 2 | 0.68 | 2.9 | (0.3–10.6) | 0.1 | (0–0.6) |
| (C) A+NLL by age group | | | | | | |
| 0–44 years | 2 | 0.16 | 12.4[a] | (1.4–45.1) | 0.3 | (0.1–1.1) |
| 45–59 years | 4 | 0.51 | 7.9[b] | (2.1–20.1) | 0.4 | (0.1–1.0) |
| 60+ years | 3 | 0.63 | 4.8[a] | (1.0–13.9) | 0.4 | (0.1–1.4) |
| (D) A+NLL by interval from treatment | | | | | | |
| 1–4 years | 5 | 0.46 | 10.9[c] | (3.5–25.4) | 0.5 | (0.4–3.0) |
| 5–9 years | 1 | 0.39 | 2.6 | (0.1–14.3) | 0.1 | (0.1–2.1) |
| 10+ years | 3 | 0.45 | 6.7[a] | (1.3–19.5) | 0.4 | (0.2–3.2) |
| (E) A+NLL following any CT | | | | | | |
| (i) By interval | | | | | | |
| 1–4 years | 2 | 0.10 | 20.0[b] | (2.3–72.2) | 1.0 | (0.1–3.7) |
| 5–9 years | 1 | 0.05 | 20.0[a] | (0.3–111.3) | 1.0 | (0.0–5.8) |
| 10+ years | 2 | 0.02 | 100.0[c] | (11.2–361.1) | 5.5 | (0.6–20.1) |
| Total | 5 | 0.18 | 27.8[c] | (9.0–64.8) | 1.5 | (0.5–3.5) |
| (ii) By age | | | | | | |
| 0–44 years | 1 | 0.02 | 50.0[a] | (0.7–278.2) | 1.4 | (0.0–8.0) |
| 45–59 years | 2 | 0.07 | 28.6[b] | (3.2–103.2) | 1.2 | (0.1–4.3) |
| 60+ years | 2 | 0.09 | 16.0[b] | (2.5–80.2) | 2.1 | (0.2–8.0) |

*EMR, excess morbidity rate (per $10^3$ PYR); [a]$P<0.05$; [b]$P<0.01$; [c]$P<0.001$.

**Table VI** Ovarian cancer: subsequent cancers of the breast in relation to age at first primary and treatment groups

| *Age group (years)* | *Treatment* RT/CT *O* | *E* | *O/E* | OT *O* | *E* | *O/E* | Total *O* | *E* | *O/E* |
|---|---|---|---|---|---|---|---|---|---|
| 0–44 | 2 | 3.2 | 0.6 | 7 | 3.0 | 2.3[a] | 9 | 6.2 | 1.5 |
| | (0) | | | (2) | | | (2) | | |
| 45–59 | 17 | 9.2 | 1.8[a] | 12 | 6.8 | 1.8[a] | 29 | 16.0 | 1.8[b] |
| | (6) | | | (4) | | | (10) | | |
| 60+ | 8 | 4.7 | 1.7 | 9 | 6.3 | 1.4 | 17 | 11.0 | 1.5 |
| | (3) | | | (3) | | | (6) | | |
| Total | 27 | 17.1 | 1.6[a] | 28 | 16.1 | 1.7[b] | 55 | 33.2 | 1.7[c] |
| | (9) | | | (9) | | | (18) | | |

Numbers in parentheses are those with at least one ovary intact; [a]$P<0.05$; [b]$P<0.01$; [c]$P<0.001$.

relative risks for corpus uteri were 2.1, 2.2, 2.8 and 2.0 for RT, CT, RT+CT and OT respectively. These results suggest that the risk is similar in each treatment group and that the real risk would have been substantially higher if surgical intervention had been accounted for.

### Discussion

Although the mortality rate was high in the series as a whole, 3,300 patients survived at least one year to be at risk for subsequent primary cancers. The large number of previous and coincidental primaries, together with the 1.5-fold risk of subsequent cancers, reflect the broad spectrum of associations between cancers of the ovary and other sites. Such relationships were demonstrated in the early multiple primary studies (Schottenfeld & Berg, 1971; Schoenberg & Christine, 1974, Prior & Waterhouse, 1981*b*) involving mainly breast, corpus uteri and colon and were tentatively attributed to common hormonal factors.

**Table VII** Ovarian cancer: subsequent cancers of connective tissue, nervous system, urinary system, lung and large bowel by treatment group and interval from first primary

| *Treatment group* | *Interval (years)* 1–9 *O* | *E* | *O/E* | 10+ *O* | *E* | *O/E* |
|---|---|---|---|---|---|---|
| Urinary system | | | | | | |
| RT/CT | 4 | 1.5 | 2.7 | 1 | 0.8 | 1.3 |
| OT | 0 | 1.5 | – | 4 | 0.9 | 4.4[a] |
| Total | 4 | 3.0 | 1.3 | 5 | 1.7 | 2.9[a] |
| Connective tissue | | | | | | |
| RT/CT | 2 | 0.2 | 10.0[a] | 0 | 0.1 | – |
| OT | 1 | 0.2 | 5.0 | 1 | 0.1 | 10.0 |
| Total | 3 | 0.4 | 7.5[b] | 1 | 0.2 | 5.0 |
| Nervous system | | | | | | |
| Any RT | 3 | 0.6 | 5.0[a] | 1 | 0.2 | 5.0 |
| OT+CT | 1 | 0.7 | 1.4 | 1 | 0.3 | 3.3 |
| Total | 4 | 1.3 | 3.1[a] | 2 | 0.5 | 4.0 |
| Lung | | | | | | |
| Any RT | 1 | 2.3 | 0.4 | 5 | 1.3 | 3.8[a] |
| OT+CT | 6 | 3.3 | 1.8 | 6 | 1.6 | 3.8[b] |
| Total | 7 | 5.6 | 1.3 | 11 | 2.9 | 3.8[c] |
| Colon/rectum | | | | | | |
| RT/CT | 8 | 6.1 | 1.3 | 33 | 2.9 | 1.0 |
| OT | 14 | 6.6 | 2.1[b] | 6 | 3.9 | 1.5 |
| Total | 22 | 12.7 | 1.7[a] | 9 | 6.8 | 1.3 |

[a]$P<0.05$; [b]$P<0.01$; [c]$P<0.001$.

Later studies separated irradiated from non-irradiated patients. Increased risks associated with radiotherapy were found for endometrium and bladder (Reimer *et al.*, 1978) and for colon (Curtis *et al.*, 1985). In patients treated between 1970 and 1975 (Reimer *et al.*, 1978) a nine-fold risk of leukaemia was found in association with chemotherapy, but not radiotherapy, but in the Connecticut Registry material, which included registrations up to 1982, the risk of acute non-lymphatic leukaemia was significantly increased in both the irradiated and non-irradiated groups, although chemotherapy may have been used in both (Curtis *et al.*, 1985). The overall relative risk of subsequent primaries in our series of 1.5 was marginally higher than the 1.2 of the recent international study (Kaldor *et al.*, 1987), probably because the latter analysis was restricted to the first subsequent primary only. The difference in relative risks was not, however, significant ($\chi_1^2 = 2.63$). In general the results from our series are consistent with previous studies with regard to the range of associations but a relationship with a specific treatment is not supported in every instance.

*Acute and non-lymphocytic leukaemia*

The emergence of A+NLL as a high risk site in later calendar years supports the growing evidence of a leukaemogenic effect of chemotherapy, in particular of the alkylating drugs. In our series a significant excess of A+NLL was found only in those treated by chemotherapy. The small but not significant increase in those treated by radiotherapy is consistent with that found for cervical cancer patients in the International Study of Cervix Cancer (Day & Boice, 1983) who had also received pelvic irradiation, particularly external irradiation (Boice *et al.*, 1987). No excess of leukaemia was found in the Birmingham series of cervical cancer patients, which formed part of the International Study, because, possibly, a higher proportion were treated solely by intracavitary radium, external irradiation being reserved for patients with disseminated disease (Prior & Brown, 1983).

The lack of effect in the RT+CT group might weigh against a treatment effect for the individual modalities. However, only a small group (609 patients) received both treatments of whom only 16.3% survived 5 years. It seems likely, therefore, that in this group the treatments were given, either together or at an interval, to palliate progressive disease and that doses would have been relatively lower and of shorter duration.

In a recent review of 83 reported cases of A+NLL following ovarian cancer (De Gramont *et al.*, 1986), all but two patients had received at least one alkylating drug; melphalan (40%), cyclophosphamide (18%), thiotepa (16%), chlorambucil (16%), 5-fluorouracil (16%) and treosulphan (14.5%), the mean duration of chemotherapy being 31.4 months (range 2–90 months) and the mean interval between cancers being 57.3 months (range 15–143 months). The five patients in our series who developed A+NLL had received cyclophosphamide, trenimon and thiotepa. The average duration of therapy (48 months) and the mean interval between ovarian cancer and A+NLL (60 months) were consistent with the above report. Our results are also consistent with the general finding in the collaborative registry study (Kaldor *et al.*, 1987) where the overall risk of A+NLL was 3.4 (after exclusion of our results) compared with 7.0 (95% CI 3.2–13.2) in our series and the risk was found to remain high beyond 10 years of follow-up. Both the EMR of 2.4 per $10^3$ PYR and the cumulative risk of 3.1% of A+NLL in our series of ovarian cancer patients were marginally higher than those found for Hodgkin's disease treated by chemotherapy, 1.0 per $10^3$ PYR and 1.7% respectively (Prior & Pope, 1988). This difference may arise because, although treatment for Hodgkin's disease is aggressive, the cyclical nature of the dosage may allow some bone marrow recovery whereas, in general, chemotherapy for ovarian cancer was continuous and of long duration. The two cases of the A+NLL found in the OT group, although not constituting a significant excess, gave a RR of 2.5. However, it was found that the patient developing A+NLL within 2 years of the ovarian cancer had previously been treated by splenectomy for aplastic anaemia and may, therefore, have been predisposed to leukaemia (Van Leeuwen *et al.*, 1987). The second case developed acute monocytic leukaemia some 22 years after the ovarian cancer when the expected number was approximately 1.0, and may, therefore, represent a chance effect.

*Breast*

Pelvic irradiation might expose the breast to a small dose of around 30 rad (Stovall, 1983) and be a potential source of increased risk, whereas ablation of the ovaries might decrease the risk. In a similar study of cervical cancer such a reduction was shown mainly for women irradiated before the age of 40 years, although the risk was reduced across all age-groups (Day & Boice, 1983). In a case–control study of this same material, however, no dose relationship could be demonstrated for breast (Boice *et al.*, 1987). Although a general consensus supports a hormonal relationship between cancers of the breast and ovary, the mechanism is not clear. The fact that the majority of patients in our series did not have a functioning ovary suggests that the increased risk of breast cancer was not directly due to the action of ovarian hormones but to other common aetiological factors. The overall relative risk in our series (1.7, 95% CI 1.3–2.2) was marginally higher than that of 1.4 found in the international study (Kaldor *et al.*, 1987). This difference is probably due to the inclusion of third and later primaries in the analysis.

*Urinary system*

An increased risk of bladder cancer has been reported after pelvic irradiation for benign disease (Wagoner, 1984) and for cancer of the cervix (Day & Boice, 1983). Data from the atomic bomb survivors (Preston, 1987) and from cervical cancer patients surviving 10 or more years (Boice *et al.*, 1987) are suggestive of a radiation dose response for bladder cancer. There is also evidence that alkylating agents, in particular cyclophosphamide, are associated with bladder cancer (Kinlen, 1981; IARC, 1981). Cyclophosphamide was a commonly used drug in our series but an RT/CT effect was not supported by our data, the excess being mainly in the OT group. There were, however, relatively few long-term survivors in the RT/CT group.

*Connective tissue*

Although the excess of soft tissue sarcomas occurred mainly in the RT/CT groups 1–9 years after first treatment, the results were not conclusive. The one case in the OT group at 10+ years had in fact been treated for breast cancer 27 years previously but radiotherapy for this condition could not be confirmed. Similar excesses of connective tissue cancers were found in the collaborative registry studies of cervical cancer (Day & Boice, 1983) and of ovarian and testicular cancer (Kaldor *et al.*, 1987) but, again, no clear distinction with type of treatment could be made.

*Nervous system*

Radiation doses to the brain from pelvic irradiation are likely to be low, probably less than 10 rads (Stovall, 1983). A marginal exccess of cancers was found in the RT group (O=4, E=0.8, RR=5.0, $P<0.01$) the excess risk, again, occurring mainly in the first nine years of follow-up, but with a generalised elevation of risk. Five of the cancers observed occurred in various lobes of the brain and were of varying histological type including one meningioma; the sixth cancer was a meningioma of the spinal cord. In the collaborative registry study a small excess of nervous system tumours was found in the ovarian cohort (Kaldor *et al.*, 1987) but a small deficit occurred in the cohort of cervical cancer patients (Day & Boice, 1983). Deaths from cancers of the central nervous system were, however, increased in patients receiving one course of radiotherapy for ankylosing spondylitis (Darby *et al.*, 1987).

*Respiratory system*

Although lung contributes to the excess in the RT group and is in excess 10 or more years after treatment, an effect for RT is not strongly supported. Only moderate excesses of lung cancer were found in the collaborative registry study (Kaldor *et al.*, 1987) and in an individual contribution to this study (Coleman *et al.*, 1987). Although radiation has been implicated in the development of lung cancer (Smith & Doll, 1982; Kato & Schull, 1982), no definitive effect for RT was found after pelvic irradiation for cervical cancer (Day & Boice, 1983) from which the lung might receive a typical dose of around 30 rads (Stovall, 1983). Two further studies reported increased risks of lung cancer after ovarian cancer but, again, no radiation effect could be demonstrated (Reimer *et al.*, 1978; Curtis *et al.*, 1985). However, our results for lung and urinary system could be consistent with a smoking or environmental pollution effect in the series (Mattison & Thorgeirsson, 1978; Wellington *et al.*, 1979), with the lower risk in the early years of follow-up reflecting our policy of cautious registration of lung tumours occurring soon after a first primary.

### *Colon and rectum*

A relationship between cancers of the ovary and colon has been reported from many studies of multiple primary cancers (Schoenberg *et al.*, 1974; Storm & Ewertz, 1985; Curtis *et al.*, 1985; Lynge *et al.*, 1985; Hoar *et al.*, 1985). The association is apparent whichever cancer is taken as the index site. A similar relationship, but to a lesser extent, was found for ovary and rectum. The association has been variously attributed to 'hormonal' factors, low parity and diet. In the context of treatment effects it may be difficult to identify a moderate increase in risk due to radiotherapy against the general effect of a more than two-fold risk for these sites. Our results were, again, consistent with those of the international study.

### *Effects of methodology and other factors*

In general, our results support those of other series showing an association between cancers of the ovary, breast, corpus uteri and large bowel which can probably be attributed to common aetiological factors. Discrepancies in the levels of risks found in the different series may arise, in part, from differing approaches to the analysis. For example, in a previous paper on the association between ovary and breast (Prior & Waterhouse, 1981*a*), we used a method of complementary analysis (Prior & Waterhouse, 1981*b*) to explore the joint association, when it was possible to include all coincidental and subsequent diagnoses. In the collaborative registry study (Kaldor *et al.*, 1987), all coincidental and first year cancers were excluded from the observed numbers and, in addition, all patients with previous or coincidental cancers were excluded from the cohort. The effect of applying the same rules to our analysis would have been to eliminate 26 cases from the observed numbers, 15 of whom had been treated by RT. Although this approach may not invalidate the analysis in the context of treatment effects, it may bias the estimates for aetiological associations unrelated to treatment: in our series a large proportion of previous and coincidental cancers and 12 of the 26 third or later primaries were of the breast, colon, rectum and uterus, which have been shown in many analyses to be associated with ovary. Thus an arbitrary exclusion of 4% of the original cohort, which may represent a high risk sub-group, will lead to an under-estimation of risk in later years.

The collaborative study used only the first subsequent cancer in the analysis. In our study we excluded no case from the cohort on the basis of coincidental cancers but we did exclude observed and expected numbers for the first year of follow-up from the analyses. The major difference in our approach was to retain patients, who developed a second primary, in the person-years at risk. The argument against this procedure was that treatment to the second primary might invalidate any conclusions drawn with respect to the initial treatment groups. Our contention was that, with respect to solid tumours, treatment effects in particular from radiotherapy would become apparent in long-term survivors and that third and subsequent primaries may well be of more relevance in this context. If the effects from superficial irradiation to rodent ulcers can be discounted, the inclusion of third and subsequent primaries did not alter the allocation of observed cases to our originally defined treatment groups.

Other factors might, however, affect the accuracy of the treatment groupings. It was found that if treatment to a primary, previous to or coincidental with the ovarian cancer, was taken into account, only 20 patients would need to be re-classified and the effect, if discernible, would enhance the risks in the OT and CT groups. Only one observed case was in doubt: a patient with a third primary of connective tissue mentioned above.

Incomplete reporting of treatments could also affect the allocation to treatment groups but it is not possible to validate the data for this without referring back to hospital records. Transcription errors of the data might also occur, but on the basis of a 4% sample we concluded that these would not have an appreciable effect on the expected numbers.

Despite some of the uncertainties inherent in routinely collected Registry data, our analyses have been able to demonstrate an increased risk of A+NLL in patients receiving chemotherapy. Whether we have seriously underestimated the risk from radiotherapy can only be resolved by further follow-up or more detailed case–control studies. However, the results do suggest that analyses by treatment group, even in the broad categories used here, enhance the use of Registry data in exploratory cohort studies.

The Multiple Primary Cancer Study was supported by the Cancer Research Campaign.

### References

BOICE, J.D., JR., BLETTNER, M., KLEINERMAN, R.A. & 36 others (1987). Radiation dose and leukaemia risk in patients treated for cancer of the cervix. *J. Natl Cancer Inst.*, **79**, 1295.

COLEMAN, M.P., BELL, C.M.J. & FRAZER, P. (1987). Second primary malignancy after Hodgkin's disease, ovarian cancer and cancer of the testis: a population-based cohort study. *Br. J. Cancer*, **56**, 349.

COLEMAN, M., DOUGLAS, A., HERMON, C. & PETO, J. (1986). Cohort study analysis with a FORTRAN computer program. *Int. J. Epidemiol.*, **15**, 134.

CURTIS, R.E., HOOVER, R.N., KLEINERMAN, R.A. & HARVEY, E.B. (1985). Second cancer following cancer of the female genital system. *Natl Cancer Inst. Monogr.*, **68**, 113.

DARBY, S.C., DOLL, R., GILL, S.K. & SMITH, P.G. (1987). Long term mortality after a single treatment course with X-rays in patients treated for ankylosing spondylitis. *Br. J. Cancer*, **55**, 179.

DAY, N.E. & BOICE, J.D., JR (1983). Summary chapter. In *Second Cancer in Relation to Radiation Treatment for Cervical Cancer*, Day, N.E. & Boice, J.D., Jr (eds) p. 137. IARC Scientific Publications: Lyon.

DE GRAMONT, A., REMES, P., KRULIK, M. & 8 others (1986). Acute leukaemia after treatment for ovarian cancer. *Oncology*, **43**, 165.

GREENE, M.H., BOICE, J.D., JR, GREER, B.E., BLESSING, J.A. & DEMBO, A.J. (1982). Acute nonlymphocytic leukaemia after therapy with alkylating agents for ovarian cancer. *N. Engl. J. Med.*, **23**, 1416.

HOAR, S.K., WILSON, J., BLOT, W.J., McCLOUGHLIN, J.K., WINN, D.M. & KANTOR, A.F. (1985). Second cancer following cancer of the digestive system in Connecticut, 1935–82. *Natl Cancer Inst. Monogr.*, **68**, 49.

IARC (1981). *Monographs on the Evaluation of the Carcinogenic Risk of Chemicals to Humans, Vol. 26, Some Anticancer and Immunosuppressive Drugs.* IARC: Lyon.

KALDOR, J.M., DAY, N.E., BAND, P. & 11 others (1987). Second malignancies following testicular cancer, ovarian cancer and Hodgkin's disease: an international collaborative survey among cancer registries. *Int. J. Cancer*, **39**, 571.

KATO, H. & SCHULL, W.J. (1982). Studies on the mortality of A-bomb survivors 7. Mortality 1950–1978. Part I: Cancer mortality. *Radiat. Res.*, **90**, 395.

KINLEN, L.J., SHEIL, A.G.R., PETO, J. & DOLL, R. (1979). Collaborative United Kingdom–Australasian study of cancer in patients treated with immunosuppressive drugs. *Br. Med. J.*, **ii**, 1461.

KINLEN, L.J., PETO, J., DOLL, R. & SHEIL, A.G. (1981). Letter: Cancer in patients treated with immunosuppressive drugs. *Br. Med. J.*, **282**, 474.

LYNGE, E., JENSEN, O.M. & CARSTENSEN, B. (1985). Second cancer following cancer of the digestive system in Denmark, 1943–80. *Natl Cancer Inst. Monogr.*, **68**, 277.

MATTISON, D.R. & THORGEIRSSON, S.S. (1978). Smoking and industrial pollutants and their effects on the menopause and ovarian cancer. *Lancet*, **i**, 187.

PETO, R., PIKE, M.C., ARMITAGE, P. & 7 others (1977). Design and analysis of randomised clinical trials requiring prolonged observation of each patient. II Analysis and examples. *Br. J. Cancer*, **35**, 1.

PRESTON, D.L., KATO, H., KOPECY, K.J. & FUJITA, S. (1987). Studies of the mortality of A-bomb survivors 8. Cancer mortality 1950–1982. *Radiat. Res.*, **111**, 151.

PRIOR, P. & BROWN, R. (1983). Second primary cancers following invasive and *in-situ* carcinoma of the cervix uteri: Birmingham Cancer Registry. In *Second Cancer in Relation to Radiation Treatment for Cervical Cancer*, Day N.E. & Boice, J.D., Jr (eds) p. 97. IARC Scientific Publications: Lyon.

PRIOR, P. & POPE, D.J. (1988). Hodgkin's disease: subsequent primary cancers in relation to treatment. *Br. J. Cancer*, **58**, 512.

PRIOR, P. & WATERHOUSE, J.A.H. (1981*a*). Multiple primary cancers of the breast and cervix uteri: an epidemiological approach to analysis. *Br. J. Cancer*, **43**, 623.

PRIOR, P. & WATERHOUSE, J.A.H. (1981*b*). Multiple primary cancers of the breast and ovary. *Br. J. Cancer*, **44**, 628.

REIMER, R.R., HOOVER, R., FRAUMENI, J.F., JR & YOUNG, R.C. (1978). Second primary neoplasms following ovarian cancer. *J. Natl Cancer Inst.*, **61**, 1195.

ROTHMAN, K.J. & BOICE, J.D., JR (1979). *Epidemiologic Analysis with a Programmable Calculator*, p. 29. NIH Publication No. 79-1649. US Government Printing Office: Washington DC.

SCHOENBERG, B.S. & CHRISTINE, B.W. (1974). The association of neoplasms of the colon and rectum with primary malignancies at other sites. *Am. J. Proctol.*, **25**, 41.

SCHOTTENFELD, D. & BERG, J.W. (1971). Incidence of multiple primary cancers IV. Cancers of the female breast and genital organs. *J. Natl Cancer Inst.*, **46**, 161.

SMITH, P.G. & DOLL, R. (1982). Mortality among patients with ankylosing spondylitis after a single treatment course with X-rays. *Br. Med. J.*, **284**, 449.

STORM, H.H. & EWERTZ, M. (1985). Second cancer following cancer of the female genital system in Denmark, 1943–80. *Natl Cancer Inst. Monogr.*, **68**, 131.

STOVALL, M. (1983). Organ doses from radiotherapy of cancer of the uterine cervix. In *Second Cancer in Relation to Radiation Treatment for Cervical Cancer*, Day, N.E. & Boice, J.D., Jr (eds) p. 131. IARC Scientific Publications: Lyon.

VAN LEEUWEN, F.E., SOMERS, R. & HART, A.A.M. (1987). Letter: Splenectomy in Hodgkin's disease and second leukaemias. *Lancet*, **ii**, 210.

WAGONER, J.K. (1984). Leukaemia and other malignancies following radiation therapy for gynecological disorders. In *Radiation Carcinogenesis: Epidemiology and Biological Significance*, Boice, J.D., Jr & Fraumeni, J.F., Jr (eds) p. 153. Raven Press: New York.

WELLINGTON, D.G., MACDONALD, E.J. & WOLF, P.F. (1979). In *Cancer Mortality: Environmental and Ethnic Factors*, p. 116. Academic Press: New York.